# Lurasidone continuation in real-world clinical cases in Japan: retrospective study of factors associated with six-month persistence

**DOI:** 10.1186/s12991-026-00640-x

**Published:** 2026-02-15

**Authors:** Kohei Fujita, Yuko Sugimoto, Ryosuke Aratake, Takaaki Yoshimoto, Mihoko Kawai, Takahide Fukatsu, Yasuhiro Mori, Makoto Nishihara, Jun Miyata

**Affiliations:** 1 Neuropsychiatric Department, Aichi Medical University, Nagakute, 480-1195 Japan; 2 Neuropsychiatric Department, Kasadera Seichiryo Hospital, Nagoya, 457-0058 Japan; 3 Multidisciplinary Pain Center, Aichi Medical University, Nagakute, 480-1195 Japan

**Keywords:** Schizophrenia, Lurasidone, Treatment persistence, Antipsychotic switching, Pharmacological class, Japan

## Abstract

**Background:**

Treatment discontinuation remains a major challenge for patients with schizophrenia, often resulting in relapse and subsequent rehospitalization. While treatment persistence is a practical marker of real-world effectiveness, findings related to lurasidone continuation in clinical settings in Japan are limited.

**Methods:**

For this retrospective observational study, the records of 62 patients with schizophrenia who had lurasidone treatment initiated at Aichi Medical University Hospital between June 2020 and December 2024 were analyzed. Treatment persistence was defined as continuation of lurasidone for ≥ 180 days. Associations of baseline characteristics, including illness duration, prior antipsychotic class (serotonin-dopamine antagonist, SDA; dopamine partial agonist, DPA; multi-acting receptor-targeted antipsychotic, MARTA), and other clinical variables, with six-month persistence were analyzed using Kaplan-Meier survival analysis and log-rank testing.

**Results:**

The six-month treatment persistence rate was 54.8%. Patients with illness duration < 5 years showed a significantly greater rate of persistence (71.4%) as compared to those with a longer duration (46.3%) (*P* = 0.046). Persistence also varied based on prior antipsychotic class, with the rate 76.5% for SDA, 50.0% for DPA, and 35.0% for MARTA (*P* = 0.045). No statistically significant differences in six-month persistence were observed across sex, treatment setting, or lurasidone dose range.

**Conclusions:**

This is the first known study to evaluate real-world lurasidone persistence in patients in Japan. The findings indicate that both pharmacodynamic compatibility and early-phase treatment can enhance persistence. The insights obtained underscore the importance of individualized treatment planning, though require further validation by use of prospective, multi-center studies.

## Introduction

Schizophrenia is a chronic and often disabling psychiatric disorder, with long-term pharmacological treatment often required to prevent relapse and functional deterioration [1, 2]. Although numerous antipsychotic agents are available, treatment discontinuation remains a major challenge in clinical practice and frequently leads to symptom exacerbation, rehospitalization, and reduced quality of life [3, 4]. Treatment persistence, defined as the duration of continuous antipsychotic treatment, is increasingly recognized as a pragmatic and integrative outcome measure in schizophrenia research [5]. It is notable that several large-scale observational studies have found associations of greater persistence with reduced risk of relapse, rehospitalization, and mortality [6, 7]. In this regard, nationwide cohort studies presented by Tiihonen et al. identified continuous antipsychotic use as the factor most protective against adverse clinical outcomes in patients with schizophrenia [6, 7].

Unlike clinical trial endpoints, used primarily to assess efficacy or safety under controlled conditions, real-world treatment persistence reflects a broader group of influential factors, including perceived effectiveness, tolerability, treatment satisfaction, and ease of use; each of which can affect patient willingness to maintain therapy over time [8]. Given its clinical relevance, a better understanding of the determinants and correlates of treatment persistence is crucial for guiding treatment selection and optimizing individualized care. However, persistence rates have been found to vary substantially across agents and populations, thus factors underlying long-term continuation remain incompletely understood [9].

Lurasidone is a relatively new second-generation antipsychotic found to have a promising tolerability profile, with previous studies showing a lower incidence of adverse effects including sedation, weight gain, and anticholinergic symptoms including constipation [10–12]. The favorable profile is thought to be related to its low affinity for noradrenaline α₁, histamine H₁, and muscarinic M₁ receptors [13], clearly distinguishing this medication from multi-acting receptor-targeted antipsychotic (MARTA) drugs such as olanzapine and quetiapine, which have broader receptor-binding profiles and are commonly associated with such adverse effects [14]. Given its relatively benign side-effect profile, lurasidone is considered likely to be associated with higher treatment adherence and relatively good treatment persistence has been shown in results of a randomized controlled trial [12]. Additionally, that study noted that while olanzapine has been reported to show higher treatment continuation rates in clinical trials, metabolic and sedative adverse effects are frequently encountered, which may affect its overall tolerability in clinical practice. In comparison, lurasidone is associated with a more favorable side-effect profile. However, real-world evidence indicating its long-term treatment persistence remains limited, particularly in Japanese clinical settings. The present study was conducted to evaluate treatment persistence of lurasidone in patients with schizophrenia exposed to routine clinical settings, with demographic and clinical predictors of discontinuation also examined. By elucidating factors that support sustained antipsychotic use, the results may contribute to more stable, tolerable, and effective long-term treatment strategies for individuals living with schizophrenia.

## Methods

This retrospective observational study was conducted at Aichi Medical University Hospital and approved by the institutional ethics committee. The primary objective was to evaluate the six-month treatment persistence rate of lurasidone. Patients were included in the analysis if they initiated lurasidone treatment at Aichi Medical University Hospital between June 1, 2020, and December 31, 2024, had follow-up data available for at least six months, and had been diagnosed with schizophrenia by board-certified psychiatrists according to the Diagnostic and Statistical Manual of Mental Disorders, Fifth Edition, Text Revision (DSM-5-TR). Patients with treatment-resistant schizophrenia were excluded from the analysis.

Treatment persistence was defined as duration from index date (initiation of lurasidone treatment) to all-cause discontinuation. Patients who continued lurasidone for more than 180 days were defined as persistent, in accordance with a previous retrospective study conducted in Italy [15]. Treatment discontinuation dates were determined based on medical records from routine monthly follow-up visits. Patients who discontinued lurasidone before 180 days were considered events at the time of discontinuation, whereas those who continued treatment beyond 180 days were censored at day 180. Kaplan–Meier survival analysis was therefore constructed using time-to-discontinuation data within the first six months after treatment initiation.

For secondary analyses, demographic, clinical, and treatment-related data were retrospectively collected from medical records. Demographic characteristics included age and sex, while clinical and treatment characteristics included treatment setting (inpatient or outpatient), duration of illness, Brief Evaluation of Psychosis Symptom Domains (BE-PSD) score at initiation of lurasidone treatment, maintenance dose of lurasidone, and prior antipsychotic use and reason for discontinuation. BE-PSD is a standardized clinician-rated scale used for assessing symptom severity that was developed based on the five-factor model of the Positive and Negative Syndrome Scale (PANSS) [16]. Prior antipsychotics were categorized into three pharmacological classes based on their receptor-binding profiles; serotonin-dopamine antagonist (SDA), dopamine partial agonist (DPA), and MARTA, a classification based on established pharmacodynamic literature and receptor affinity data [17–19]. Lurasidone dosage was categorized as follows: low (< 40 mg/day), medium (≥ 40 to ≤ 60 mg/day), and high (> 60 to ≤ 80 mg/day).

Patient data were collected using an opt-out approach, in accordance with ethical regulations, by announcing the study on the hospital website and allowing patients to decline participation. Retrospective data were obtained from those obtained at all clinical visits following lurasidone initiation, which typically occurred monthly. To standardize the follow-up duration and avoid bias from unequal observation periods, all analyses were limited to the first six months after the index date, even for patients whose follow-up visits exceeded six months.

Continuous variables were summarized using descriptive statistics, including mean and 95% confidence interval (CI), while non-normally distributed variables, such as Duration of illness and BE-PSD scores, were summarized using median and interquartile range (IQR). Comparisons between male and female patients were performed using the t test for age and the Mann–Whitney U test for duration of illness and BE-PSD scores. Kaplan-Meier survival analysis was conducted to estimate time to treatment discontinuation, and group differences in regard to treatment persistence were evaluated using a log-rank test. Fisher’s exact test was conducted to examine the association between reason for switching and pharmacological class of the prior antipsychotic. To determine whether BE-PSD scores differed according to classification of the previous antipsychotic, a Kruskal-Wallis rank sum test was used, while a Mann-Whitney U test was performed to examine if treatment setting had an effect. All statistical analyses were performed using IBM SPSS Statistics for Windows, version 25.0 (IBM Corp., Armonk, NY, USA). A two-sided alpha level of 0.05 was considered to indicate statistically significant. No adjustment for multiple comparisons was applied, as all analyses were exploratory in nature. Adverse events and relevant medical histories were coded according to the Medical Dictionary for Regulatory Activities.

## Results

**Fig. 1:**
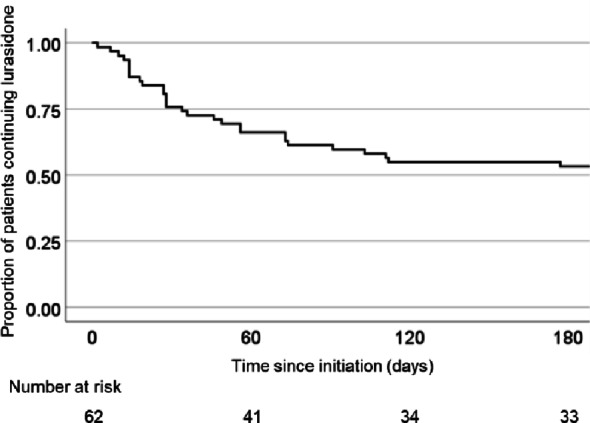
Kaplan–Meier survival curve showing lurasidone treatment persistence

**Fig. 2:**
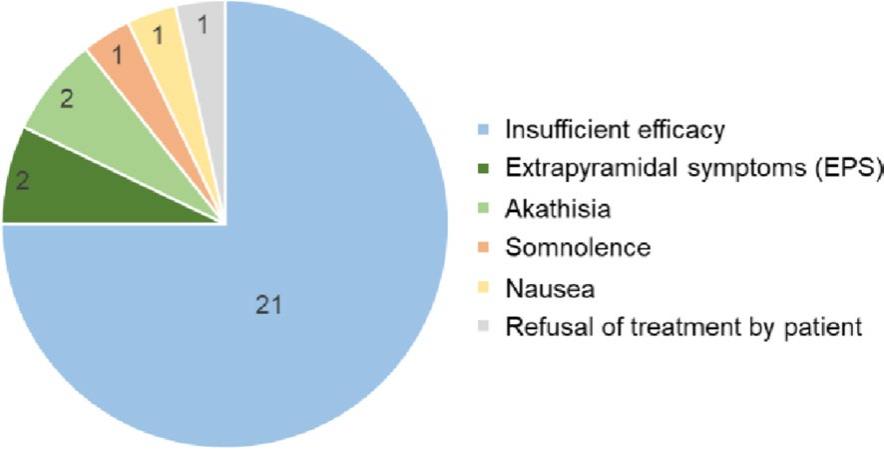
Reasons for discontinuation of lurasidone treatment among 62 patients. The most common reason was insufficient efficacy (*n* = 21, 33.9%), followed by extrapyramidal symptoms (*n* = 2, 3.2%), akathisia (*n* = 2, 3.2%), somnolence (*n* = 1, 1.6%), nausea (*n* = 1, 1.6%), and medication refusal (*n* = 1, 1.6%)

Among patients with schizophrenia receiving care at Aichi Medical University, 65 had lurasidone treatment initiated during the study period. Of these, three were referred to other medical institutions before treatment continuation could be confirmed, thus were excluded from the present analysis. Complete follow-up data were available for the remaining 62 patients (95.6%) and included in the final analysis. Baseline sociodemographic and clinical characteristics are summarized in Table 1. The mean age of the cohort was 46.9 (43.1–50.7) years, and 80.6% were female. The mean duration of illness at baseline was 13.9 (2.1–22.6) years. The mean BE-PSD scores at baseline were 11.1 (7.3–13.0) and female patients had significantly higher baseline BE-PSD scores than male patients (*P* = 0.0098). During the six-month follow-up period, the treatment persistence rate was 54.8% (Fig. 1). The most common reason for discontinuation was insufficient efficacy (*n* = 21, 33.9%), followed by extrapyramidal symptoms (*n* = 2, 3.2%), akathisia (*n* = 2, 3.2%), somnolence (*n* = 1, 1.6%), nausea (*n* = 1, 1.6%), and refusal of treatment by patient (*n* = 1, 1.6%). These factors related to treatment discontinuation are summarized in Fig. 2.

**Table 1:** Baseline sociodemographic and clinical characteristics

	All	Male	Female	*P* value
Age	46.9 (43.1–50.7)	40.8 (30.8–49.8)	48.4 (44.2–52.6)	0.11
Duration ofillness	13.9 (2.1–22.6)	11.4 (1.4–17.6)	14.5 (2.8–23.8)	0.34
BE-PSD	11.1 (7.3–13.0)	7.6 (4.0–11.0)	12.0 (9.0-15.8)	0.0098*

Age is presented as mean with 95% confidence intervals (CIs), while duration of illness and BE-PSD scores are presented as mean with interquartile range (IQR). Comparisons between male and female patients were performed using the t test for age and the Mann–Whitney U test for duration of illness and BE-PSD. A statistically significant sex difference was observed for BE-PSD scores, with higher scores in female patients than in male patients (*P* = 0.0098)

BE-PSD, Brief Evaluation of Psychosis Symptom Domains

*P < 0.05

**Fig. 3:**
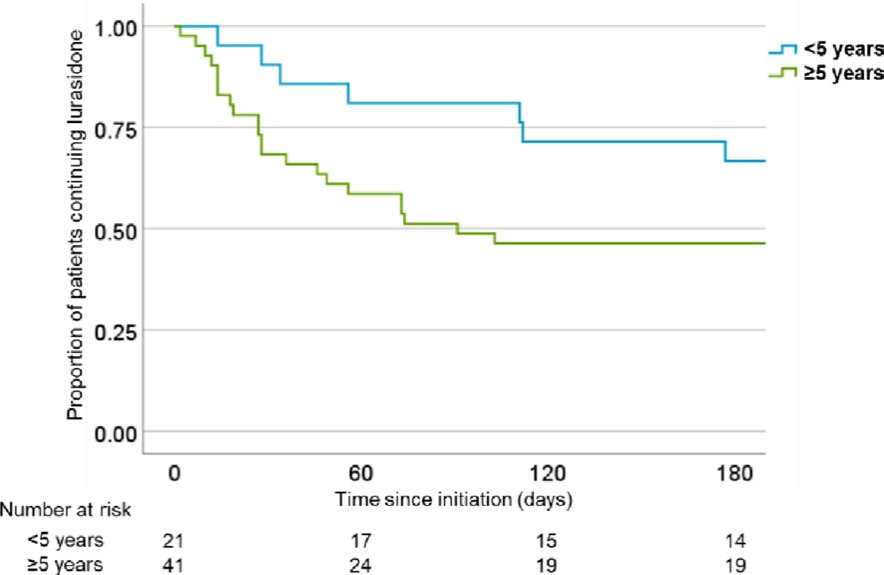
Kaplan–Meier survival curves for lurasidone treatment persistence stratified by duration of illness (< 5 vs. ≥ 5 years)

The associations of treatment persistence at six months with baseline demographic, clinical, and treatment characteristics were examined (Table 2). Analysis showed no significant difference in persistence rate between male (41.7%) and female (58.0%) patients (*P* = 0.38). Similarly, persistence did not significantly differ based on treatment setting at initiation (inpatient 55.3%, outpatient 53.3%; *P* = 0.77). In contrast, there was a significant difference based on duration of illness, as patients with a duration < 5 years showed a persistence rate of 71.4%, whereas those with a duration ≥ 5 years had a significantly lower rate of 46.3% (*P* = 0.046). A Kaplan–Meier survival curve illustrating this difference is presented in Fig. 3. As for the different initial lurasidone dose ranges, persistence rate for low dose was 44.4%, for medium dose was 52.0%, and for high dose was 60.7%, with no significant differences among the dosage groups (*P* = 0.41).

**Table 2:** Association between treatment persistence at six months and baseline demographic, clinical, and treatment characteristics

Variable	Classification	No.	Six-month persistence (%)	*P* value
Sex	Male	12	41.7 (9.0-74.4)	0.38
	Female	50	58 (43.8–72.2)	
Treatment setting	In patient	15	55.3 (40.5–70.0)	0.77
	Out patient	47	53.3 (40.5–70.1)	
Duration of illness	< 5 years	21	71.4 (50.3–92.5)	0.046*
	≥ 5 years	41	46.3 (30.4–62.2)	
Dose range of lurasidone	Low	9	44.4 (3.9–85.0)	0.41
	Medium	25	52 (30.9–73.1)	
	High	28	60.7 (41.4–80.0)	

Six-month treatment persistence is reported with 95% confidence intervals (CIs). Patients with an illness duration < 5 years had a significantly higher persistence rate (71.4%) compared with those with a longer duration (46.3%) (*P* = 0.046, log-rank test). No statistically significant differences in six-month persistence were observed across sex, treatment setting, or lurasidone dose range

SDA, serotonin-dopamine antagonist; DPA, dopamine partial agonist; MARTA, multi-acting receptor-targeted antipsychotic

*P < 0.05

**Fig. 4:**
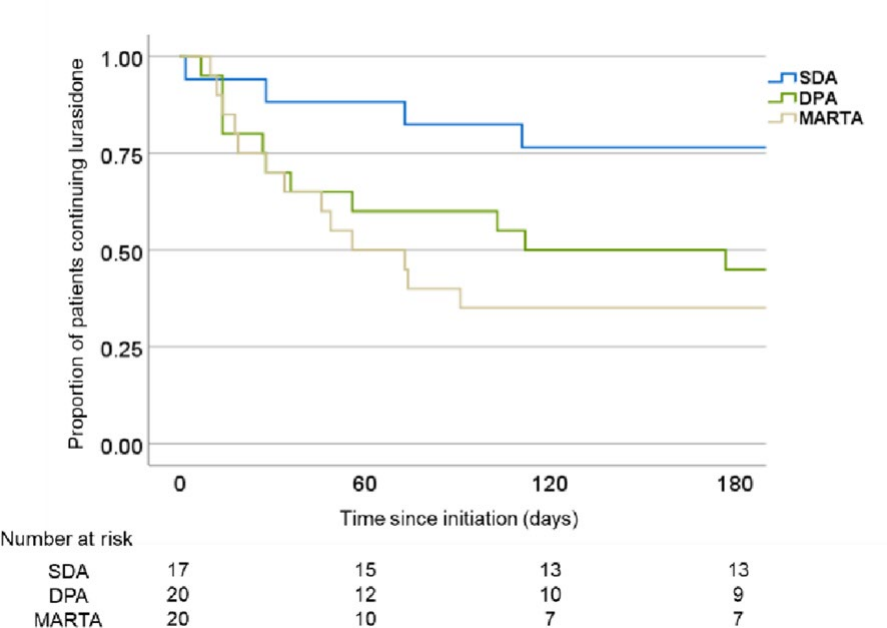
Kaplan–Meier survival curves for lurasidone treatment persistence according to prior antipsychotic class (SDA, DPA, MARTA). SDA, serotonin-dopamine antagonist; DPA, dopamine partial agonist; MARTA, multi-acting receptor-targeted antipsychotic

Persistence rates differed according to pharmacological class of prior antipsychotic, as six-month persistence was 76.5% for patients switched from SDA, 50.0% for those switched from DPA, and 35.0% for those switched from MARTA. Kaplan–Meier survival analysis showed a statistically significant difference in persistence among those (log-rank test, *P* = 0.045). These findings are visualized in Fig. 4, with the results summarized in Table 3. The method of switching from the previous antipsychotic was not specified in the records analyzed. There were significant differences among the SDA, DPA, and MARTA pharmacological classes regarding reasons for switching (*p* = 0.007). There were no significant differences for BE-PSD scores among the categories of prior antipsychotic class (*P* = 0.43). As compared to outpatients, inpatients exhibited significantly higher BE-PSD scores (*P* = 0.000014). These results are presented in Table 4. The analyses were exploratory in nature, thus no adjustments for multiple comparisons were applied.

**Table 3:** Comparisons of six-month treatment persistence rates across Pharmacological classes of prior antipsychotics

Pharmacological class	Drug name	No.	Six-month persistence (%)	*P* value (between classes)
SDA	RIS	2		
	PAL	4		
	BNS	7		
	PER	2		
Total SDA		17	76.5 (54.0–99.0)	
DPA	APZ	10		
	BPZ	10		0.045*
Total DPA		22	50 (26.0–74.0)	
MARTA	OZP	14		
	QTP	2		
	ASE	2		
Total MARTA		17	35 (12.1–57.9)	
Other	HPD	1	N/A	

Six-month treatment persistence is reported with 95% confidence intervals (CIs). P values represent differences in six-month persistence rates between pharmacological classes (SDA, DPA, MARTA), as assessed by a log-rank test. No statistical comparisons were made between individual antipsychotic drugs

SDA, serotonin-dopamine antagonist; DPA, dopamine partial agonist; MARTA, multi-acting receptor-targeted antipsychotic; RIS, risperidone; PAL, paliperidone; BNS, blonanserin; PER, perospirone; APZ, aripiprazole; BPZ, brexpiprazole; OZP, olanzapine; QTP, quetiapine; ASE, asenapine; HPD, haloperidol, N/A, not applicable

*P < 0.05

**Table 4:** Relationships between prior antipsychotic class, treatment setting, and BE-PSD

		BE-PSD	P value
Pharmacological class	SDA	12.0 (10.0–14.0)	0.43
	DPA	10.0 (6.5–13.0)	
	MARTA	10.5 (8.0-13.8)	
Treatment setting	In patient	17.0 (15.5–20.0)	0.000014*
	Out patient	10.0 (7.0–12.0)	

BE-PSD scores are reported as median (IQR). To examine whether BE-PSD scores differed according to classification of previous antipsychotic and/or treatment setting, Kruskal–Wallis rank sum and Mann–Whitney U tests, respectively, were performed

SDA, serotonin-dopamine antagonist; DPA, dopamine partial agonist; MARTA, multi-acting receptor-targeted antipsychotic; BE-PSD, brief evaluation of psychosis symptom domains

*P < 0.05

## Discussion

In this retrospective observational study of patients with schizophrenia, the six-month treatment persistence rate for lurasidone was found to be 54.8%, which compares favorably with previous studies, such as the CATIE trial that examined the effectiveness of antipsychotic medications in real-world settings [3]. However, as compared to a real-world study conducted in Italy that reported a higher six-month persistence rate of 68.4% [15], the present findings suggest a somewhat lower continuation rate. One possible explanation for this difference may be disparities in available dosing ranges among countries. Osborne et al. (2018) reported that a higher dosage of lurasidone was associated with greater treatment persistence in routine clinical settings [20]. In Japan, lurasidone is approved up to 80 mg/day, whereas other countries have higher doses (up to 160 mg/day) available for administration, a limitation that may have contributed to the somewhat lower persistence observed in the present study. Additionally, no statistically significant differences in persistence were observed across the initial dose groups in the present cohort, possibly reflecting the relatively narrow dosing range available in Japan. Beyond dosing, discrepancies among related studies may also be explained by differences in design and patient populations. For example, Osborne et al. conducted a 12-month follow-up investigation, while persistence after six months was evaluated in the present study. Moreover, their cohort included not only patients with schizophrenia, but also those with schizoaffective and bipolar disorders, who may have different treatment trajectories and tolerability profiles. However, the proportions of reported adverse events were largely consistent with those in the present study. In support of this notion, a retrospective study conducted in the United States reported a six-month discontinuation rate of 44.4%, comparable to that observed in the present study [21]. Although the actual dosage of lurasidone was not specified in that study and interpretation of the findings should be made with caution, they imply that factors other than dosage may also contribute to treatment persistence.

Notably, treatment persistence in the present cohort was significantly higher in patients with an illness duration < 5 years as compared to those with a longer period. This finding supports the therapeutic advantages of early intervention for patients with schizophrenia, consistent with the “critical period hypothesis,” which posits that a window exists early in the disease course during which treatment is more likely to produce a favorable long-term outcome [22]. This hypothesis has been supported by several biological studies. Longitudinal neuroimaging studies have shown that brain volume decreases with increasing illness duration [23], and that progressive gray matter loss is most pronounced during the first-episode phase [24], after which the rate of change attenuates and approaches that of normal aging [25]. These structural changes are associated with worsening negative symptoms and declines in neuropsychological functioning [26]. Furthermore, microglial activation and alterations in neuroplasticity have been implicated in both acute exacerbations and progressive disease processes in schizophrenia [27], and may be related to variability in antipsychotic treatment responsiveness [28]. Treatment persistence is known to be influenced by antipsychotic medication efficacy, the incidence of adverse effects, and the degree of functional improvement [29], and patients in the early phase of schizophrenia have been reported to show better treatment response and functional outcomes than those in chronic stages [2, 30]. Taken together, these neurobiological and clinical features of early-stage schizophrenia may help to account for the higher treatment persistence observed in patients with shorter illness duration. However, because data on treatment response and functional improvement were not available in the present study, further research using detailed clinical assessments is required.

In addition to the critical period hypothesis, psychosocial factors may also contribute to the higher treatment persistence observed in patients with a shorter illness duration. Insight, which is known to be associated with medication adherence and functional outcomes [31], is a dynamic psychological process in early psychosis [32] and tends to be more stable in first-episode patients than in chronic stages [33]. These features may have facilitated sustained treatment engagement in patients within five years of illness onset. Nevertheless, greater insight has also been linked to depressive symptoms and hopelessness, indicating that its overall impact on outcomes is complex [31].

From a social and clinical perspective, irrespective of whether specialized early intervention programs are formally implemented, the importance of close clinical follow-up and supportive care during the early phase of psychosis has been widely recognized [34]. Systematic reviews of early intervention in psychosis services have emphasized the value of sustained clinician contact, continuity of care, and supportive engagement in improving treatment outcomes [34]. Such patterns of care in the early stage of illness may have further facilitated treatment persistence in patients with a shorter duration of schizophrenia.

A noteworthy finding was the significant associations of pharmacological class with prior antipsychotic use and also subsequent treatment persistence. As compared to patients switched from DPA or MARTA, those switched from SDA exhibited the highest six-month persistence rate. Although previous meta-analysis results have demonstrated differences in efficacy and tolerability across antipsychotic classes [11], few studies have examined whether these distinctions can be translated into differences in real-world treatment continuation. From a pharmacological perspective, the present findings should be interpreted with consideration of the potential influence of differences in anticholinergic activity. For example, when switching from an agent with a strong anticholinergic property, such as olanzapine or quetiapine, to lurasidone, which has minimal anticholinergic effects, patients may develop anticholinergic withdrawal symptoms, including nausea, headache, and vomiting [35]. In such cases, instead of a direct switch, an approach such as continuation with slow titration should be considered to help mitigate withdrawal symptoms and potentially improve treatment persistence. Nevertheless, details regarding switching strategies, such as add-on or cross-titration, were not documented in the present analyzed data, though are important to consider. Such strategies have particular clinical relevance when considering pharmacological differences between a previously administered antipsychotic and lurasidone. Accordingly, in light of these considerations, the present findings should be interpreted with caution.

Osborne et al. (2018) reported no significant differences in treatment persistence between patients switched from olanzapine and those switched from other another antipsychotic [20]. However, their study included a broader range of psychiatric diagnoses, including schizoaffective and bipolar disorders, which may limit direct comparability with the present schizophrenia-only cases. Future studies focused exclusively on patients with bipolar disorder may help to elucidate disorder-specific differences.

Interestingly, not only did treatment persistence vary significantly based on pharmacological class of prior antipsychotic administration, but the reasons for switching also differed across these classes. Specifically, patients switched from a DPA agent were more likely to have discontinued their prior medications due to insufficient efficacy, whereas those switched from an SDA or MARTA agent showed a more balanced distribution of switching reasons, including issues related to efficacy and tolerability. Previous studies have suggested that patients who switch antipsychotics due to tolerability issues tend to exhibit higher treatment continuation, likely because the new medication provides immediate relief from adverse effects and enhances overall treatment acceptance [20]. Thus, the comparatively lower persistence rate following the switch from DPA to lurasidone, relative to SDA, may reflect a greater frequency of switching prompted by insufficient efficacy.

It should also be noted that treatment persistence has been reported to be lower in patients with treatment-resistant schizophrenia [15]. However, such patients were not included in the present study. In the present cohort, BE-PSD scores were significantly higher for inpatients as compared to outpatients, suggesting that the results appropriately reflected symptom severity. In addition, baseline BE-PSD scores differed significantly by sex, with higher scores observed in female patients. Nevertheless, there was no significant difference in treatment persistence between inpatients and outpatients, nor between male and female patients. Moreover, no differences in BE-PSD scores among the prior antipsychotic classes were observed. Taken together, these findings suggest that prior antipsychotic class was more closely associated with treatment persistence than baseline symptom severity in the present subjects.

## Limitations

There are several limitations that should be acknowledged. First, the retrospective and observational design precludes causal inference between treatment persistence and clinical or pharmacological factors. While associations were identified, confounding variables not analyzed may have influenced the results. Second, the sample size was relatively small, particularly after dividing into subgroups based on prior antipsychotic class or illness duration. This limited statistical power for detecting subtle effects and precluded use of multivariate modeling, such as Cox proportional hazards regression, to control for potential confounders. Third, the generalizability of our findings may be limited, as the study was conducted using data from patients treated at a single university hospital in Japan. It is likely that clinical practice elements, patient demographics, and prescription determination patterns differ in other institutions and countries. Furthermore, 80.6% of the present subjects were female, thus the findings may not be fully representative of the broader population of patients with schizophrenia and caution should be exercised when generalizing the results. Moreover, the maximum approved dose of lurasidone in Japan (80 mg/day) is lower as compared to that in some other countries (e.g., up to 160 mg/day), which could have an influence on dose-related treatment outcomes and limit comparability of findings obtained in other studies. Fourth, reasons for switching of antipsychotic medication and treatment discontinuation were extracted from medical records, and may have been subject to clinician interpretation or incomplete documentation. Furthermore, while symptom severity was assessed using the BE-PSD, other patient-related factors, such as insight, treatment attitudes, or psychosocial functioning, were not evaluated, despite their potential relevance to treatment continuation. Finally, since no correction for multiple comparisons was applied during analysis, as this study was exploratory in nature, the risk of Type I error cannot be ruled out and the findings should be interpreted with caution. Prospective studies using a larger number of cases and multi-center data, as well as comprehensive assessments of patient characteristics are warranted to confirm and expand upon these preliminary results.

## Conclusions

This study represents the first real-world investigation of lurasidone treatment persistence in patients in Japan, and the findings highlight the potential impact of pharmacological class of prior antipsychotics and duration of illness on treatment continuation. Greater persistence was observed for patients who switched from SDA agents as well as those in the early phase of illness, suggesting that pharmacodynamic compatibility and clinical staging have important roles for sustaining treatment. These findings underscore the need for individualized treatment strategies and warrant large-scale, prospective studies for further validation.

## Data Availability

The datasets used and/or analyzed during the current study are available from the corresponding author on reasonable request.

## Abbreviations

MARTA: Multi-acting receptor-targeted antipsychotic
BE-PSD: Brief Evaluation of Psychosis Symptom Domains
PANSS: Positive and Negative Syndrome Scale
SDA: Serotonin-dopamine antagonist
DPA: Dopamine partial agonist
CI: Confidence interval
IQR: Interquartile range
